# A multimodal submillimeter MRI atlas of the human cerebellum

**DOI:** 10.1038/s41598-024-55412-y

**Published:** 2024-03-07

**Authors:** Wenjiao Lyu, Ye Wu, Khoi Minh Huynh, Sahar Ahmad, Pew-Thian Yap

**Affiliations:** 1 Department of Radiology, University of North Carolina, Chapel Hill, NC USA; 2 Biomedical Research Imaging Center, University of North Carolina, Chapel Hill, NC USA

**Keywords:** Neuroscience, Neurology

## Abstract

The human cerebellum is engaged in a broad array of tasks related to motor coordination, cognition, language, attention, memory, and emotional regulation. A detailed cerebellar atlas can facilitate the investigation of the structural and functional organization of the cerebellum. However, existing cerebellar atlases are typically limited to a single imaging modality with insufficient characterization of tissue properties. Here, we introduce a multifaceted cerebellar atlas based on high-resolution multimodal MRI, facilitating the understanding of the neurodevelopment and neurodegeneration of the cerebellum based on cortical morphology, tissue microstructure, and intra-cerebellar and cerebello-cerebral connectivity.

## Introduction

The cerebellum, also known as the ‘little brain’, comprises approximately 80% of the brain’s neurons despite accounting for only 10% of the total brain volume^1^. The cerebellum’s total surface area is about 78% that of the neocortex^2^, owing to the compact and elaborate folding pattern of the cerebellar cortex. The cerebellum is the fastest growing structure during infancy^3^ and plays a pivotal role in motor and nonmotor functions involving cognitive and emotional processes^4–7^. Due to its association with functions beyond motor activities, it is conjectured to be implicated in multiple brain disorders^8–11^.

A detailed atlas is essential for investigating cerebellar morphology and function. There were several attempts in constructing cerebellar atlases based on in vivo T1-weighted (T1w) and T2-weighted (T2w) MR images. However, this endeavor remains challenging since the cerebellar cortex is tightly folded^12^ with barely distinguishable boundaries between gray matter (GM) and white matter (WM) in folia and fissures, hence the current cerebellum atlases tend to oversimplify the structure of the cerebellum by simply classifying the corpus medullare (CM) as white matter and the rest of the cerebellum as gray matter^13–16^.

Here, we provide a multifaceted cerebellar atlas constructed based on open-access high-resolution multimodal MRI data^17^. The atlas covers the anatomical parcellation, cortical surfaces, microstructure, and fiber tracts of the cerebellum, including

- A semi-automatic parcellated anatomical cerebellar atlas with both WM and GM meticulously delineated;
- Surface meshes encapsulating the outer and inner surfaces;
- Fiber streamlines characterizing intra-cerebellar and cerebello-cerebral structural connectivity; and
- Maps of tissue microstructure generated using submillimeter diffusion MRI.

We expect that our cerebellar atlas will facilitate generating new insights into the role of the cerebellum and its relationship with the cerebrum.

## Results

### The anatomical atlas delineates regional cerebellar gray matter and white matter

**Figure 1:**
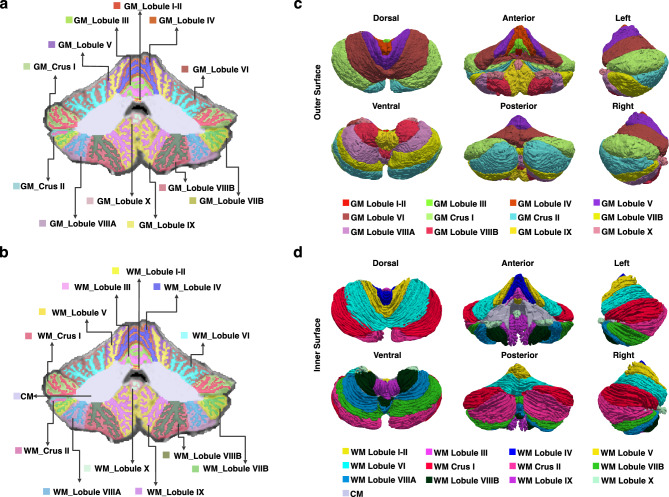
Volumetric (**a** and **b**) and surface (**c** and **d**) atlases of the cerebellum. WM: White matter; GM: Gray matter; CM: Corpus medullare.

Each cerebellar hemisphere was parcellated into CM, Lobule I–II, Lobule III, Lobule IV, Lobule V, Lobule VI, Crus I, Crus II, Lobule VIIB, Lobule VIIIA, Lobule VIIIB, Lobule IX, and Lobule X. In line with Schmahmann’s principle^18^, Lobules I–II, III, IV, and V belong to the anterior lobe, Lobule VI, Crus I, Crus II, Lobule VIIB, Lobule VIIIA, Lobule VIIIB, and Lobule IX belong to the posterior lobe, and Lobule X is also known as the flocculus. Except the CM, the GM and WM of each cerebellar subregion were delineated, resulting in a cerebellar atlas comprising 12 GM regions, 12 WM regions, and 1 CM region in each hemisphere (Fig. 1a–b). In addition to adhering to fissures in demarcating cerebellar subregions, our atlas follows high anatomical fidelity in delineating cerebellar GM and WM.

### The surface atlas captures complex folding patterns

**Figure 2:**
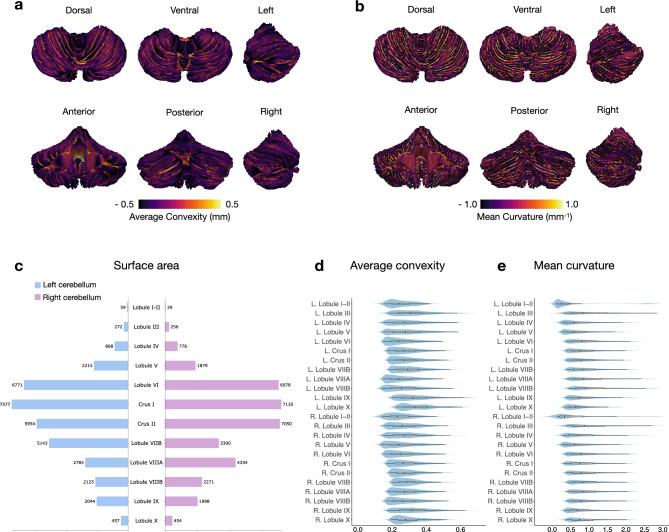
Features of the cerebellar inner surface: (**a**) average convexity (mm), (**b**) mean curvature (mm^−1^), (**c**) regional surface area (mm^2^), (**d**) average convexity (mm, absolute value, top 50%), and, (**e**) mean curvature (mm^-1^, absolute value, top 50%). The box within each violin plot marks the interquartile range and median.

We reconstructed the cortical surfaces of the cerebellum, including the outer surface (GM hull) (Fig. 1c) and the inner surface (WM-GM interface) (Fig. 1d). Average convexity (Fig. 2a) captures coarse-scale folding patterns at the lobule level, whereas mean curvature (Fig. 2b) captures fine-scale folding patterns of folia and fissures. Both coarse and fine folding patterns are preserved in the reconstructed cerebellar surfaces. The inner surface area of cerebellum is 72,740 mm^2^, which is about 38.82% of the cerebral inner surface (182,680 mm^2^) of the same subject. Crus I is the largest lobule in terms of area in both hemispheres and the aggregate size of Lobule VI, Crus I, and Crus II accounts for 57% of the cerebellar inner surface area (Table 1 and Fig. 2). The area of right Crus II is larger than the left, consistent with previous studies^15^. The areas of left Lobule VIIB and right Lobule VIIIA are substantially larger than their contralateral counterparts, owing to the varying amount of folia^15^. Areas of the other lobules are similar across hemispheres. Furthermore, the Lobules IX and X in the left hemisphere exhibit higher median convexity compared to the other lobules. Lobule I-II shows the minimum median curvature and Lobule VIIIB shows the largest median curvature in both hemispheres. Additionally, we observed that the median curvature values in the anterior lobe are relatively smaller than those in the posterior lobe.

**Table 1:** Regional features of the inner surfaces of the left (L) and right (R) cerebellar hemispheres. Average convexity and mean curvature are shown in the form of median (lower quartile, upper quartile).

Region	Surface area	Average Convexity	Mean Curvature
	(mm^2^)	(mm, absolute value)	(mm^-1^, absolute value)
L. Lobule I–II	59	0.154 (0.065, 0.240)	0.134 (0.080, 0.265)
L. Lobule III	272	0.175 (0.066, 0.306)	0.333 (0.156, 0.627)
L. Lobule IV	868	0.152 (0.065, 0.274)	0.267 (0.116, 0.519)
L. Lobule V	2215	0.151 (0.077, 0.280)	0.278 (0.110, 0.572)
L. Lobule VI	6771	0.151 (0.074, 0.243)	0.374 (0.152, 0.717)
L. Crus I	7577	0.172 (0.085, 0.272)	0.381 (0.155, 0.708)
L. Crus II	5954	0.180 (0.094, 0.275)	0.389 (0.156, 0.730)
L. Lobule VIIB	5143	0.184 (0.098, 0.292)	0.391 (0.156, 0.739)
L. Lobule VIIIA	2785	0.149 (0.072, 0.243)	0.400 (0.169, 0.733)
L. Lobule VIIIB	2125	0.143 (0.065, 0.262)	0.436 (0.200, 0.755)
L. Lobule IX	2044	0.199 (0.090, 0.320)	0.398 (0.173, 0.696)
L. Lobule X	457	0.224 (0.114, 0.334)	0.368 (0.183, 0.638)
R. Lobule I–II	39	0.138 (0.069, 0.231)	0.133 (0.070, 0.266)
R. Lobule III	256	0.157 (0.073, 0.260)	0.400 (0.173, 0.693)
R. Lobule IV	776	0.147 (0.054, 0.271)	0.315 (0.139, 0.616)
R. Lobule V	1879	0.139 (0.062, 0.242)	0.276 (0.120, 0.565)
R. Lobule VI	6978	0.152 (0.076, 0.254)	0.353 (0.144, 0.704)
R. Crus I	7135	0.169 (0.083, 0.267)	0.328 (0.119, 0.691)
R. Crus II	7050	0.186 (0.095, 0.275)	0.379 (0.150, 0.718)
R. Lobule VIIB	3300	0.198 (0.105, 0.286)	0.401 (0.159, 0.751)
R. Lobule VIIIA	4334	0.175 (0.088, 0.274)	0.410 (0.175, 0.756)
R. Lobule VIIIB	2271	0.164 (0.082, 0.262)	0.410 (0.184, 0.721)
R. Lobule IX	1998	0.174 (0.077, 0.314)	0.360 (0.162, 0.633)
R. Lobule X	454	0.193 (0.104, 0.280)	0.390 (0.176, 0.682)
Total	72740	0.169 (0.083, 0.271)	0.370 (0.150, 0.705)

### The microstructure atlas maps spatial cellular distribution

**Figure 3:**
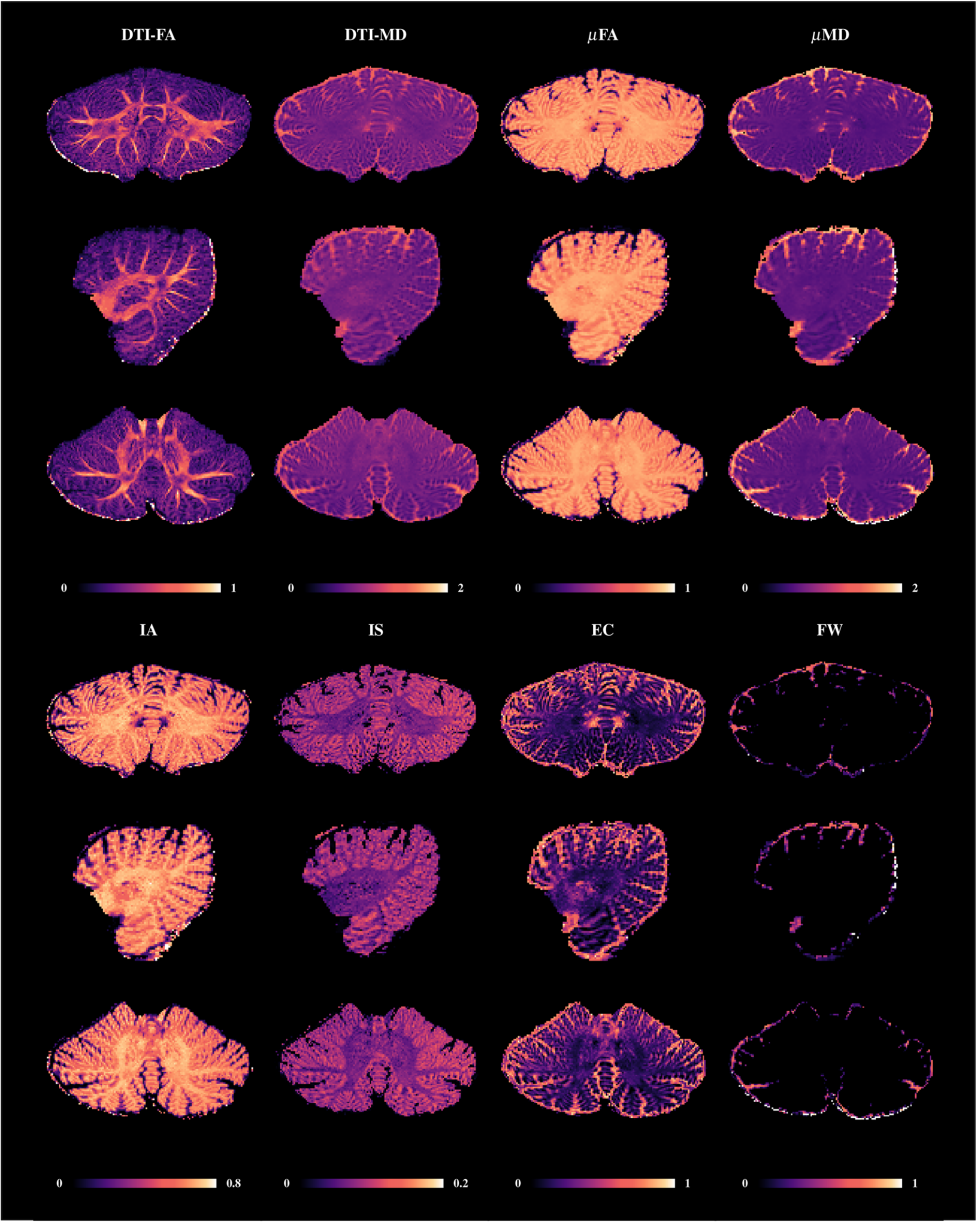
Cerebellar microstructure. Diffusion-based cerebellar microstructure indices: fractional anisotropy (DTI-FA), microscopic FA ($\mu$FA), mean diffusivity (DTI-MD, μm^2^ ms^−1^), microscopic MD ($\mu$MD, μm^2^ ms^−1^), and intra-axonal (IA), intra-soma (IS), extra-cellular (EC), and free-water (FW) volume fractions.

Using submillimeter diffusion MRI (dMRI), we characterize cerebellar tissue microstructure via a technique called spherical mean spectrum imaging (SMSI)^19^ (Fig. 3). The high-resolution SMSI maps show clear delineation of WM, GM, and cerebrospinal fluid (CSF). The fractional anisotropy (FA) map delineates deep WM structure with non-homogeneous spatial pattern in the CM, highlighting the deep cerebellar nuclei. These nuclei are undulate circular GM structures with lower FA values^20,21^. This visibility of the deep cerebellar nuclei is often obscured due to low T1w contrast or partial volume effects in dMRI with typically lower resolutions^22,23^. The microscopic FA ($\mu$FA) and intra-axonal volume fraction (IAVF) highlight the unique arbor vitae branching pattern of cerebellar WM^24^, consisting of mossy and climbing fibers going from the brainstem to the cerebellar cortex^25^. The intra-soma (IS) map highlights the cerebellar GM with organized and densely-packed granule cells and Purkinje cell bodies^25,26^. The large number of cell bodies, with typically lower packing density than axons, contained in the cerebellar GM, including Purkinje cells, Granule cells, Stellate cells, Basket cells, and Golgi cells^25,27^, result in a greater intercellular space compared with the cerebellar WM. This is corroborated by the high extra-cellular volume fraction (ECVF) observed in the cerebellar cortex. Similarly high ECVF values can be observed for the deep cerebellar nuclear mass. The mean diffusivity (MD), microscopic mean diffusivity ($\mu$MD), and free water (FW) maps provide further insights into the microstructural organization of the cerebellum without distinguishing intra- and extra-cellular water diffusion^28–30^.

**Figure 4:**
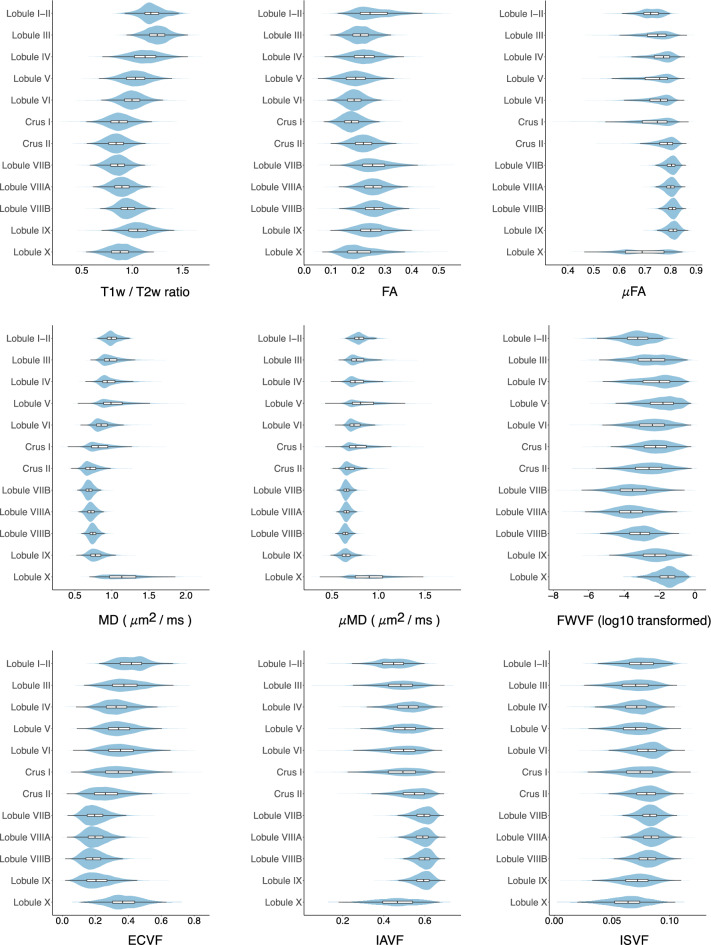
The gradients of regional T1w/T2w ratio, FA, MD, and SMSI index averages. The box within each violin plot marks the interquartile range and median.

**Figure 5:**
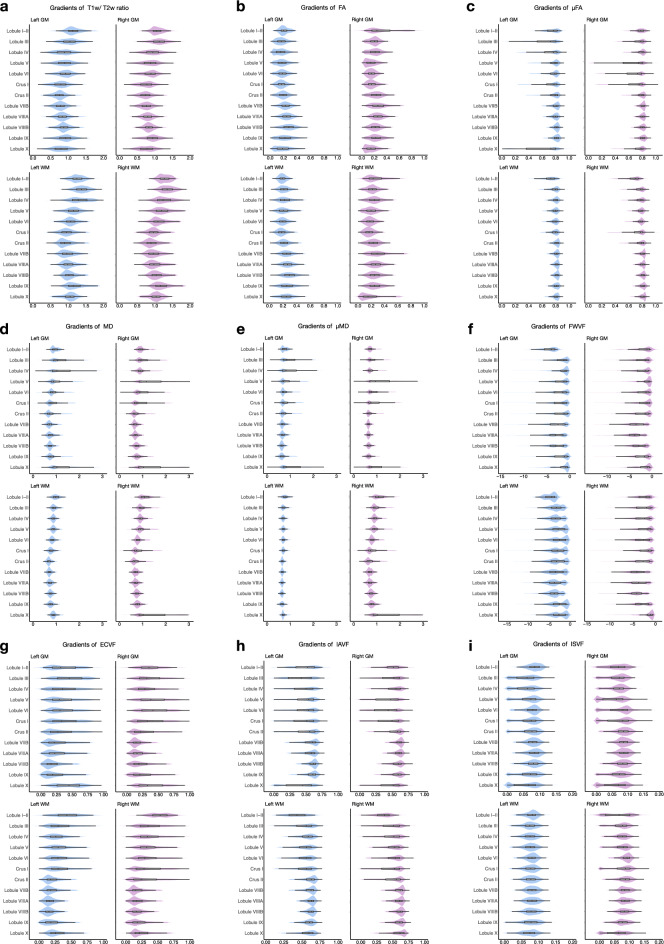
The gradients of regional averages for GM (top) and WM (bottom) of the left (light blue) and right (light violet) hemispheres: (**a**) T1w/ T2w ratio, (**b**) fractional anisotropy (DTI-FA), (**c**) microscopic FA ($\mu$FA), (**d**) mean diffusivity (DTI-MD, μm^2^ ms^−1^), (**e**) microscopic MD ($\mu$MD, μm^2^ ms^−1^), (**f**) free-water volume fraction (FWVF, in log10), (**g**) extra-cellular volume fraction (ECVF), (**h**) intra-axonal volume fraction (IAVF), and, (**i**) intra-soma volume fraction (ISVF) . The box within each violin plot marks the interquartile range and median.

We calculated the T1w/T2w ratios, as well as FA, MD, and SMSI values for all voxels in the cerebellum (Fig. 4). Our results reveal an S-shaped pattern of median T1w/T2w ratios in cerebellar subregions, with relatively small values in Crus II and Lobule X, consistent with previous findings^31^. Moreover, we observed an intriguing pattern with Crus I and Lobule X demonstrating reduced values for FA, $\mu$FA, and IAVF, and elevated values for MD, $\mu$MD, and ECVF. Low values of T1w/T2w ratios, FA, $\mu$FA, and IAVF are commonly considered indicative of inadequate myelination, as are high values of MD, $\mu$MD, and ECVF. Additionally, we present a comprehensive comparison of the aforementioned parameters between GM and WM in different cerebellar hemispheres within each lobule (Fig. 5).

### The tract atlas reflects connectivity within the cerebellum and with the cerebrum

**Figure 6:**
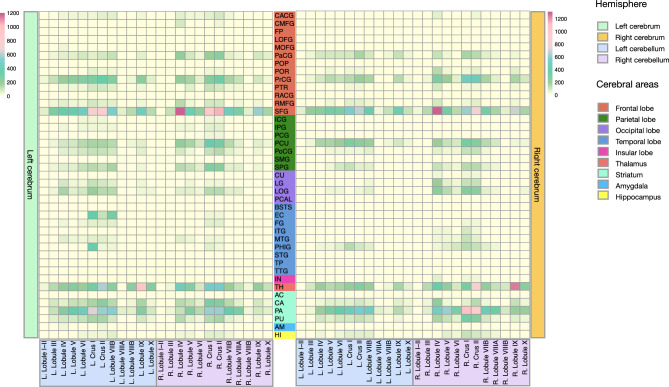
Connectivity matrices between the cerebellum and the cerebrum weighted by the sum of streamline weights (SSWs). CACG: caudal anterior cingulate gyrus; CMFG: caudal middle frontal gyrus; FP: frontal pole; LOFG: lateral orbitofrontal gyrus; MOFG: medial orbitofrontal gyrus; PaCG: paracentral lobule; POP: pars opercularis; POR: pars orbitalis; PrCG: precentral gyrus; PTR: pars triangularis; RACG: rostral anterior cingulate gyrus; RMFG: rostral middle frontal gyrus; SFG : superior frontal gyrus; ICG: isthmus cingulate gyrus; IPG: inferior parietal gyrus; PCG: posterior cingulate gyrus; PCU: precuneus; PoCG: postcentral gyrus; SMG: supramarginal gyrus; SPG: superior parietal gyrus; CU: cuneus; LG: lingual gyrus; LOG: lateral occipital gyrus; PCAL: pericalcarine; BSTS: banks of the superior temporal sulcus; EC: entorhinal cortex; FG: fusiform gyrus; ITG: inferior temporal gyrus; MTG: middle temporal gyrus; PHIG: parahippocampal gyrus; STG: superior temporal gyrus; TP: temporal pole; TTG: transverse temporal gyrus; IN: insula; TH: thalamus; AC: accumbens area; CA: caudate; PA: pallidum; PU: putamen; AM: amygdala; HI: hippocampus.

**Figure 7:**
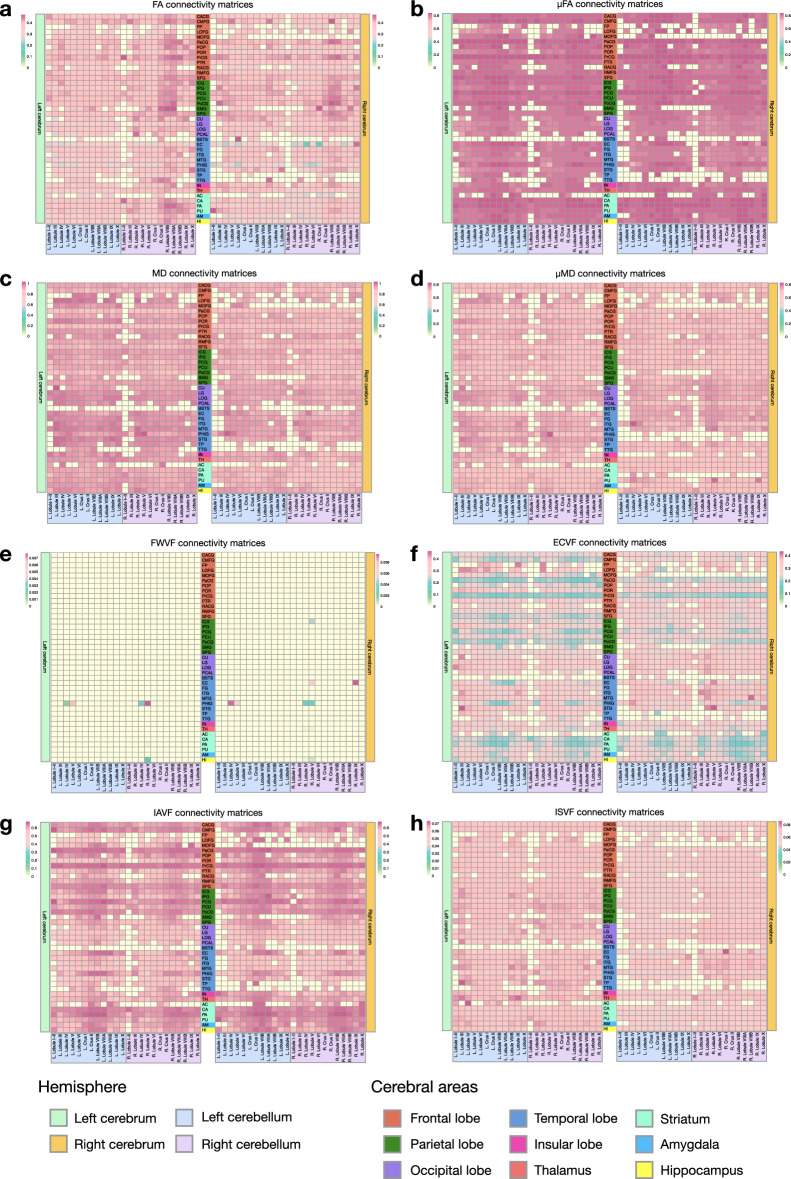
Connectivity matrices between the cerebellum and the cerebrum weighted by, (**a**) fractional anisotropy (FA), (**b**) microscopic FA ($\mu$FA), (**c**) mean diffusivity (MD, μm^2^ ms^−1^), (**d**) microscopic MD ($\mu$MD, μm^2^ ms^−1^), (**e**) free-water volume fraction (FWVF), (**f**) extra-cellular volume fraction (ECVF), (**g**) intra-axonal volume fraction (IAVF), and, (**h**) intra-soma volume fraction (ISVF).

**Figure 8:**
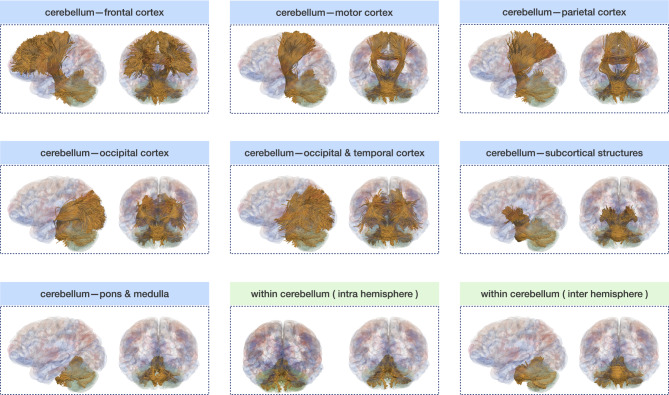
Tract bundles within the cerebellum (green) and between the cerebellum and the cerebrum (blue).

We investigated the connectivity of each cerebellar subregion with the cerebrum (Fig. 6). Our results indicate that the cerebellum is highly connected with the frontal, parietal, temporal, occipital, and subcortical regions of the cerebrum. Additionally, it is noteworthy that there is strong connectivity between bilateral cerebellar subregions, such as Lobule IV, Crus I, and Crus II, with cerebral subregions, including the superior frontal gyrus, precentral gyrus, paracentral lobule, superior parietal gyrus, precuneus, lateral occipital gyrus, caudate, pallidum, and thalamus. We also quantified cerebello-cerebral connectivity using SMSI (Fig. 7). The results indicate higher FA and IAVF values, as well as lower MD, $\mu$MD, and ECVF values, for connections between the cerebellum and the cerebral motor areas, such as the precentral gyrus, paracentral lobule, and postcentral gyrus, in comparison to those between the cerebellum and cerebral regions associated with cognitive functions. This contrast is particularly prominent for connectivity based on ECVF (Fig. 7f) and IAVF (Fig. 7g). We identified fiber bundles that traverse the cerebellum to different regions of the cerebrum (Fig. 8), including 13 bundles to the frontal cortex, 6 bundles to the motor cortex, 3 bundles to the parietal cortex, 3 bundles to the occipital cortex, 3 bundles to the occipital and temporal cortices, 12 bundles to the subcortical structures, and 9 bundles to the pons and medulla. Additionally, we identified multiple tracts within the cerebellum and classified them based on whether they spanned both cerebellar hemispheres.

## Discussion

In this work, we constructed using T1- and T2-weighted and diffusion MRI a high-resolution multimodal atlas that captures morphological, microstructural, and connectivity features of the cerebellum. We introduced for the first time a cerebellar parcellation consisting of 24 distinct GM and WM regions in each hemisphere, in addition to the CM. We calculated the connectivity between cerebellar subregions and with the cerebrum, providing evidence supporting the involvement of the cerebellum in cognitive functions. Our high-resolution cerebellar parcellation map can contribute to the fine-grained investigation of the evolution of cerebellar GM and WM during neurodevelopment and neurodegeneration.

Recent methods for cerebellar segmentation, such as SUIT^13^ and CERES^16^, rely on T1-weighted images and do not consider information on connectivity and microstructure provided by diffusion MRI. These methods remain coarse in their delineation of within-lobule WM and GM and fail to distinguish smaller regions associated with Lobule I to Lobule IV. Unlike existing cerebellar parcellations, we utilized a semi-automatic cerebellar parcellation method with submillimeter structural and diffusion MRI to generate a comprehensive cerebellar atlas that distinguishes WM and GM at the folium level and at the same time allows quantification of cerebellar tissue microstructure and intra-cerebellar and cerebello-cerebral connectivity. We referred to Schmahmann’s nomenclature^18^ in segmenting the cerebellum into CM, Lobule I–II, Lobule III, Lobule IV, Lobule V, Lobule VI, Crus I, Crus II, Lobule VIIB, Lobule VIIIA, Lobule VIIIB, Lobule IX, and Lobule X. Our cerebellar anatomical atlas not only delineates all the cerebellar subregions, but also further divides each cerebellar subregion into GM and WM, providing more details for a closer investigation into the cerebellum. The cerebellar atlas generated by our parcellation method can assist researchers in analyzing the cerebellar cortex and in investigating lobular WM/GM changes.

The considerably larger surface area and intricately folded pattern of the human cerebellar cortex, in comparison to other mammals^32,33^, indicate its potential relevance to cognitive performance. Recent years have witnessed growing interest in the morphology of the human cerebellar cortex^34–36^. Our surface atlas allows further insight into cerebellar folding complexity. Interestingly, we found that the surface ratio between the cerebellum and cerebrum is 38.82%, which is lower than 78% reported by Sereno et al.^2^ and 68% reported by Zheng et al.^34^. This discrepancy might be caused by the relatively lower spatial resolution of our data. Additionally, it is worth noting that the ratio computed by Sereno et al.^2^ is not based on the cerebral surface area of the same individual^2^. Zheng et al.^34^ made the assumption that the shrinkage coefficients of the cerebrum and the cerebellum are identical, potentially leading to a biased ratio.

Methods such as diffusion tensor imaging (DTI)^37^, diffusion kurtosis imaging (DKI)^38^, neurite orientation dispersion and density imaging (NODDI)^39^ have been used to characterize the tissue microstructure of the cerebral WM. With a typical 3-layer configuration^40^, the average thickness of cerebellar cortex is around 1.2 mm^34^, which is only about 1/3 of the cerebral cortex^41^, causing considerable challenge in tissue-specific microstructural analysis. By leveraging SMSI, we constructed an atlas incorporating $\mu$FA, $\mu$MD, and IA, IS, EC, and FW volume fractions in addition to regularly used indices such as FA and MD, enabling more comprehensive assessment of WM and GM tissue microstructure.

Mildly myelinated cortical regions are often associated with high-order cognitive function^42^, and intracortical circuit complexity is inversely associated with myelination^43^. Therefore, myelination is hypothesized to potentially impede synaptic plasticity, which is a crucial neurobiological foundation for memory and learning^43^. Our analysis involving T1w/T2w ratio, FA, MD, and SMSI indices indicated that Crus I, Crus II, and Lobule X exhibited lower myelination compared with other subregions. These observations are in line with previous studies supporting the roles of Crus I and Crus II in working memory and Lobule X in emotional function^44,45^. Furthermore, our results showed connections with increased myelination between the cerebellum and motor areas of the cerebrum, as indicated by higher IAVF and lower ECVF, compared with connections to regions responsible for higher-order functions.

A growing number of studies have focused on the role of cerebellum in cognitive processes^4^, including attention^46^, working memory, learning ability, executive function, language processing^47^, and emotional experience^6,7^. In this study, we borrowed ideas from the concept of co-segmentation^48^ and designed a multi-scale voxel clustering method driven by voxel-wise connectivity profiles for cerebellar parcellation. We calculated the connectivity between each subregion of the cerebellum and the cerebrum, and observed that there is significant connectivity between the cerebellum and cerebral regions such as the superior frontal gyrus, superior parietal gyrus, precentral gyrus, paracentral lobule, precuneus, lingual gyrus, lateral occipital gyrus, hippocampus, parahippocampal gyrus, caudate nucleus, pallidum, and thalamas. The thalamus and striatum might be pivotal components in cerebello-cortical circuit as previous studies have well documented^49–52^. Our findings also revealed connectivity between the cerebellum and the motor cortex, which is consistent with the widespread recognition of the cerebellum as a crucial hinge in modulating motor coordination^53,54^. It is especially intriguing to find that the majority of cerebral regions connected with cerebellar regions are responsible for cognitive processes. The superior frontal gyrus is thought to be associated with higher cognitive function, high levels of working memory, and emotional function^55,56^ in addition to being involved in sensorimotor integration processes^57^. The superior parietal lobule has been shown to be associated with working memory^58^, spatial cognitive function^59^, and attention shift^60^. The precuneus has been well documented to be involved in high-order cognitive functions such as first-person perspective experience^61^, episodic source memory retrieval^62^, memory-related imagery, and visual imagery^63^. The lingual gyrus is regarded as an important area related to memory^64,65^, depression^66,67^, and global shape processing when reading^68^. The hippocampus has been widely considered as a key structure involved in memory^69–71^. The parahippocampus is considered to be involved in memory function, attention and visuospatial processing^72,73^. The lateral occipital cortex is regarded as a critical structure for object recognition^74^. Furthermore, employing fiber tracking with submillimeter dMRI data, we constructed an atlas of fiber tracts connecting the cerebellum with the cerebral cortex, subcortex (including the thalamus and striatum), and pons and medulla, as well as fiber tracts connecting cerebellar subregions. Our study provides evidence supporting the role of the cerebellum in cognitive processes.

There are varying opinions on the functional organization of the cerebellum. One view is that the functional map of the cerebellum is coupled to the anatomical map. Studies have shown that there is a gradient change in function from “motor” to “cognition” and then to “motor” from cerebellar Lobule I to Lobule VII (including Crus I, Crus II, and Lobule VIIb), and then to Lobule IX^10,44,45^. Some studies summarized that the medial, intermediate, and lateral zones of the cerebellum are functionally associated with emotional, motor, and cognitive tasks, respectively^6,75^. Another view is that the functional boundaries do not match the lobular boundaries^76^, and that functional differences between cerebellar lobules may not be greater than the functional differences within lobules. Our findings demonstrate that each lobule of the cerebellum, especially Crus I, Crus II, and Lobule VIIb, is connected with multiple cerebral regions, supporting the functional complexity of the cerebellum. Our study provides evidence for the diversity of cerebellar functions across lobules.

White matter fiber tracts from various directions often pass through deep nuclei within the CM of the cerebellum, presenting great challenges for fiber tracking. Therefore, we chose to ignore fiber tracts that originate from or terminate in the CM, albeit at the risk of losing some potentially meaningful results. Another limitation of diffusion tractography is its inability in differentiating between afferent and efferent fibers tracts. Our atlas is based on a single participant and further work is needed to propagate information captured in our atlas to a larger population.

## Conclusion

To sum up, our cerebellar atlas captures the fine-scale folding patterns of folia and fissures, allowing the investigation of cortical morphology with respect to cerebellar functions. Our atlas is enriched by information on tissue microstructure, allowing fine-grained characterization of subvoxel tissue compartments. Our atlas also captures information on cerebellar and cerebello-cerebral connectivity, facilitating the understanding of the role of the cerebellum in functions such as motor coordination and cognition.

## Methods

### Dataset and preprocessing

MRI data^17^ was acquired at submillimeter isotropic resolutions for a young Caucasian male using the MGH-USC 3T Connectom scanner with a custom-built 64-channel coil. Scanning was carried out in 9 two-hour sessions. A custom built 64-channel coil with a personalized motion-robust head stabilizer was used to improve image quality and minimize motion artifacts^77–80^. The submillimeter resolution provides additional anatomical details important for smaller structures such as the cerebellum. The acquisition parameters are as follows:

- T1-weighted (T1w) image: repetition time (TR) = 2530 ms; echo time (TE) = 1.29, 3.08, 4.83, 6.58 ms; inversion time (TI) = 1110 ms; isotropic resolution = 0.7 mm; flip angle = $7^\circ$; FOV = $256 \times 256 \times 146\,\text {mm}^3$; total acquisition time = 8.38 minutes.
- T2-weighted (T2w) image: TR = 3200 ms; TE = 563 ms; isotropic resolution = 0.7 mm; FOV = $224 \times 224 \times 180\,\text {mm}^3$; total acquisition time = 8.4 minutes.
- Diffusion-weighted images: TR = 3500 ms; TE = 75 ms; in-plane resolution=0.76 mm; FOV = $220 \times 288\,\text {mm}^2$; b-values = 0, 1000, 2500 s mm^−2^ for 144, 420, and 840 directions, respectively. Total acquisition time was approximately 14.5 hours.

The reconstructed diffusion data was denoised with MP-PCA^81^, corrected for signal drift^82^, susceptibility-induced distortion, eddy currents distortion, signal dropoff, motion artifact, and gradient nonlinearity using FSL^83^ and MRtrix3^84^. Finally, visual QC was performed. These are common preprocessing steps widely used in diffusion MRI to ensure high quality data^85^. Finally, T1- and T2-weighted images were transformed into diffusion space via multi-contrast nonlinear registration using ANTS.

Utilizing ITK-SNAP^86^, WM was initially outlined, followed by the identification and annotation of the CM located centrally in the bilateral cerebellar hemispheres. Annotation was performed slice by slice on the T1w image and subsequently checked on coronal, sagittal, and transverse sections to ensure anatomical continuity and no isolated voxels. The annotation was further refined based on the T2w image.

Based on automated tract-based parcellation, we performed manual editing to further subdivide the cerebellum into subregions based on cerebellar fissures, following the principles established by Schmahmann et al.^18^. Specifically, we initially delineated Lobule V and Lobule VI using the primary fissure, which serves as the boundary between the anterior and posterior lobes of the cerebellum. Subsequently, we identified the posterolateral fissure as the boundary between Lobule IX and Lobule X, which also demarcates the posterior lobe from the flocculus. Several other significant fissures were used for further delineation, including the superior posterior fissure to delineate Lobule VI and Crus I, the horizontal fissure to separate Crus I and Crus II, the ansoparamedian fissure to separate Crus II and Lobule VIIB, the intrabiventer fissure to distinguish Lobule VIIIA and Lobule VIIIB, and the secondary fissure to separate Lobule VIIIB and Lobule IX.

### Surface construction

The GM-WM boundaries are carefully identified, particularly in regions with micro-foliations. The inner surface at the GM-WM interface was reconstructed, and its average convexity and mean curvature were computed using FreeSurfer^87^ to characterize folding patterns.

### Spherical mean spectrum imaging

Cerebellar microstructure was quantified using SMSI^19,88,89^. Specifically, SMSI involves a diffusion spectrum that encompasses all biologically plausible diffusion processes at fine to coarse scales, including both anisotropic and isotropic diffusion. SMSI assesses the contributions of diffusion in micro-environments to the voxel signal. The volume fractions of various compartments, such as free-water, intra-soma, intra-axonal, and extra-cellular diffusion, along with microscopic fractional anisotropy and mean diffusivity, were calculated. The utilization of the spherical mean ensures that SMSI measurements are free from the confounding effect of fiber orientation.

Implementation details are described in^19,88^. Briefly, the spectrum is spanned by anisotropic atoms with longitudinal diffusivity $\lambda _{\parallel }$ from $1.0\, \times \,10^{-3}\hbox {mm}^{2}\,\hbox {s}^{-1}$ to $2.0\,\times \,10^{-3}\hbox {mm}^{2}\,\hbox {s}^{-1}$ and perpendicular diffusivity $\lambda _{\perp }$ satisfying $\frac{\lambda _{\parallel }}{\lambda _{\perp }} \ge 1.1$ as well as isotropic atoms with diffusivity from $0\,\times \,10^{-3}\hbox {mm}^{2}\,\hbox {s}^{-1}$ to $3.0\,\times \,10^{-3}\hbox {mm}^{2}\,\hbox {s}^{-1}$, in step size $0.1\,\times \,10^{-3}\hbox {mm}^{2}\,\hbox {s}^{-1}$. By considering the whole diffusion spectrum biologically feasible for the human brain, SMSI does not rely on hypothetical compartment diffusivity and cardinality, unlike other microstructure models. This is useful for studying lesser investigated structures like the cerebellum.

### Cerebellar parcellation

Our parcellation method involves (i) cerebellar tractography^90,91^; (ii) streamline clustering^92^; (iii) voxel annotation based on streamline clusters^93^; (iv) sparse non-negative matrix factorization (NMF) for cerebellparcellation^93^; and (v) manual refinement.

Tractography was performed using asymmetric fiber orientation distribution functions (AFODFs) to more accurately represent complex axonal patterns (e.g., bending, fanning, and crossing) and alleviate gyral bias to promote better cortico-cortical connectivity^91^. Fiber streamlines were generated by successively following the local directions determined from the AFODFs^91^. The active cortex tractography (ACT)^90^ technique was employed to facilitate the penetration of streamlines into the cortex through the superficial WM. ACT uses a scouting mechanism to aid sharper turns into the cortical gyrus. This feature is particularly beneficial for the cerebellum, which has dense cortical folds. Whole-brain tractography was carried out with 64 random seeds per voxel, yielding approximately 100 million streamlines, but only streamlines that terminated at the cerebellar cortical regions were retained. We employed a recent approach^93^ to identify a total of $M=4,8,12,16,20,24,28,32,36,38$ parcels based on $B=50,100,150,200,250,300,350,400,450,500$ bundles determined by unsupervised clustering of the cerebellar tractography streamlines, resulting in $10 \times 10$ initial label maps.

Multi-scale consistent parcellation was performed by jointly considering all the initial label maps. First, we encoded the relationships between the 100 initial label maps and *N* voxels using a matrix $C=(c_{ki})$, where $c_{ki}$ is the label of the *i*-th voxel in the *k*-th label map. Next, we decomposed the encoding matrix into $\bar{M}$ components using sparse NMF, yielding a component matrix *H* and the corresponding component coefficients. Each voxel was assigned to the component that corresponds to the largest coefficient in its coefficient vector, resulting in a parcellation map *S* with $\bar{M}$ parcels. We corrected for isolated voxels in *S* by incorporating information from neighboring voxels^93^. We manually determined the best parcellation with $\bar{M}=25$ from a range of candidate parcellations ($\bar{M}=4$ to $\bar{M}=100$), based on previous studies^14,16,94^. Finally, we manually inspected and refined the segmentation according to an existing protocol for hierarchical parcellation of the cerebellum^15,18,95^.

### Connectome construction

Each connection was quantified via the weighted streamline count (SC) between two regions. Diffusion tractography may not always yield accurate connectivity between brain regions due to various factors^96^. To mitigate this problem, we assigned to each streamline a weight that was determined by tractogram matching with fixel-wise fiber densities using SIFT2^97^. We further weighted the connections by streamline-averaged microstructural measurements (FA, MD, and SMSI indices)^98–101^ to reflect microstructural integrity^96,102,103^.

### Statistical analysis and visualization

The dplyr^104^ package in R^105^ (version 3.8.0, https://www.R-project.org/) was utilized to remove outlier surface features, T1w/ T2w ratios, and SMSI indices. The cleaned data were then visualized using the ggplot2 package^106^ in R. The pheatmap package^107^ in R was employed to display the connectivity matrices. ITK-SNAP^86^ was utilized to visualize the volumetric and surface atlases. ParaView (version 5.10.1, https://www.paraview.org/) was used to visualize the average convexity and mean curvature of the cerebellar inner surface as well as the fiber bundles.

## Data Availability

The original T1-weighted, T2-weighted, and diffusion MRI data is freely available at https://doi.org/10.5061/dryad.nzs7h44q2 and https://doi.org/10.5061/dryad.rjdfn2z8g^17^. The multimodal cerebellar atlas is available at https://cerebellum.yaplab.io: Data/T1w_Space: Surface and volumetric data in T1w space. Data/DWI_Space: Surface, volumetric, and tract data in the DWI space. Data/Numerical/Microstructure: Voxel-wise microstructural measurements for each cerebellar subregion. Data/Numerical/Connectivity: Intra-cerebellar and cerebello-cerebral connectivity. Data/Cerebral_Regions.csv: Name abbreviations of cerebral regions. Data/Cerebellar_Regions.csv: Label indices of cerebellar regions.
